# Development of the blood–brain barrier

**DOI:** 10.1242/dev.205134

**Published:** 2026-01-23

**Authors:** Benjamin D. Gastfriend, Richard Daneman

**Affiliations:** Departments of Neurosciences and Pharmacology, University of California, San Diego, La Jolla, CA 92093, USA

**Keywords:** Blood–brain barrier, Central nervous system, Vasculature, Wnt/β-catenin signaling, Endothelial cells, Pericytes, Astrocytes

## Abstract

The blood–brain barrier (BBB) is a term used to describe the specialized properties of vasculature in the central nervous system (CNS) that regulate the exchange of ions, molecules and cells, thereby maintaining CNS homeostasis. These BBB properties are largely possessed by vascular endothelial cells, which exhibit molecular specializations that distinguish them from endothelial cells in other organs. BBB development – orchestrated by complex interactions between endothelial cells, neural progenitor cells, pericytes, astrocytes, neurons and other cell types that form the neurovascular unit – is initiated concurrently with CNS angiogenesis and continues during expansion and maturation of the vasculature. Importantly, the Wnt/β-catenin signaling pathway plays a central role in barriergenesis, the initial acquisition of BBB phenotype in endothelial cells. In this Review, we summarize current understanding of BBB development, including timing and underlying cellular and molecular mechanisms. We also discuss established and emerging techniques for studying BBB development and highlight important unanswered questions in the field.

## Teaser

**Summary:** A Review of the development of the blood–brain barrier, including timing, underlying cellular and molecular mechanisms, established and emerging tools and techniques, and important unanswered questions.

## Introduction

**Fig. 1.**
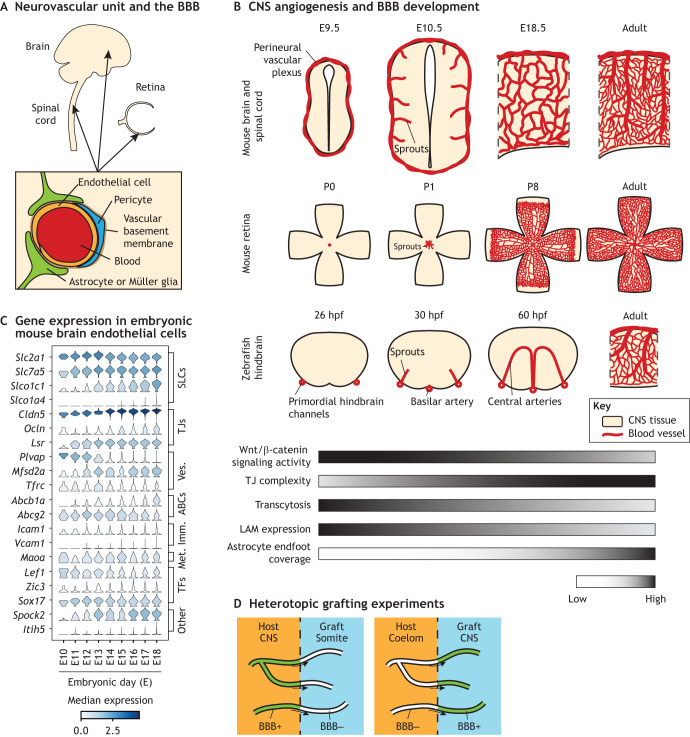
**Development of blood–brain barrier properties and control by extrinsic factors.** (A) Schematic of the neurovascular unit and blood–brain barrier (BBB). (B) Time course of central nervous system (CNS) angiogenesis and BBB development in mice and zebrafish. Schematics of retina vasculature represent the superficial layer and are based on the development of the superficial vascular plexus in mouse retinas shown in Stahl et al. (2010). E, embryonic day; hpf, hours post-fertilization; LAM, leukocyte adhesion molecule; P, postnatal day; TJ, tight junction. (C) Acquisition of the BBB transcriptional profile during mouse embryogenesis. Single cell RNA-seq data were re-analyzed from La Manno et al. (2021). The complete gene expression and metadata matrix was obtained from the authors' website (http://mousebrain.org/development/downloads.html). Endothelial cell clusters (as annotated by the authors but excluding cluster Vendo4 because of enrichment for pericyte markers) at the embryonic timepoints shown were subset and used to visualize BBB gene expression. Analysis was performed in Scanpy v1.11.2 (Wolf et al., 2018). ABCs, ATP-binding cassette transporters; Imm., immune-related; Met., metabolism-related; SLCs, solute carriers; TJs, tight junctions; TFs, transcription factors; Ves., vesicle trafficking-related. (D) Schematic summary of the heterotopic grafting experiments performed by Stewart and Wiley (Stewart and Wiley, 1981).

The blood–brain barrier (BBB) is a term used to describe the specialized vasculature in the central nervous system (CNS) that tightly regulates the exchange of ions, molecules and cells, and thus maintains a precisely regulated extracellular environment necessary for neural electrochemical signaling (Stern and Gautier, 1921). The BBB exists across the CNS, including in the brain, spinal cord, retina and optic nerve (Fig. 1A). Early evidence of a BBB comes from experiments where parenterally administered water-soluble dyes do not stain the brain or spinal cord, suggesting that mechanisms exist to exclude blood-borne substances from the CNS (Goldmann, 1909; Wislocki, 1920). Eventually, other studies demonstrated that the retina, which is part of the CNS, also exhibits low dye uptake, extending the concept of a BBB to all CNS tissues (Ashton, 1965; Cunha-Vaz et al., 1966; Shakib and Cunha-Vaz, 1966). Furthermore, electron microscopy studies showed that the vascular endothelium, and not the glia limitans (see Glossary, Box 1), forms a barrier to injected molecular tracers in tetrapods and most other vertebrate species (Brightman and Reese, 1969; Bundgaard and Abbott, 2008; Reese and Karnovsky, 1967).

Box 1. Glossary **Arachnoid barrier.** A layer of epithelial-like cells in the middle layer of the meninges (the arachnoid mater) that forms a barrier between the outer dura mater, which is outside the CNS, and the underlying subarachnoid space containing cerebrospinal fluid.

**Arteriovenous axis.** The anatomical dimension along which blood pressure varies, i.e. artery – arteriole – capillary – venule – vein. Vasculature exhibits functional and molecular differences along the arteriovenous axis, a phenomenon called zonation.

**Blood–retina barrier (BRB).** The set of specialized properties of retinal vasculature that is directly analogous to the blood–brain barrier (BBB). The BRB has frequently been used as a model system for studying BBB development because retinal angiogenesis occurs postnatally, and the vascular tree, from arterioles to capillaries to venules, is highly stereotyped and can be simultaneously visualized. In this Review, we use the term BRB to refer to the inner BRB. An outer BRB (between choroid and retina) is formed by the retinal pigment epithelium.

**Circumventricular organ (CVO).** A region of the CNS that carries out neurosecretory or neurosensory function; vasculature in CVOs lack a blood–brain barrier.

**Experimental autoimmune encephalomyelitis (EAE).** An animal model of demyelinating autoimmune disease typically induced by immunizing animals with myelin antigens or by introducing myelin-reactive T cells. EAE is commonly used as a model of multiple sclerosis.

**Fenestra.** A small pore in a vascular endothelial cell that serves as a conduit between the blood and tissue.

**Glia limitans.** A layer of astrocyte endfeet. The glia limitans perivascularis surrounds blood vessels in the CNS parenchyma; the glia limitans superficialis exists at the interface between CNS parenchyma and meninges.

**Intraparenchymal vessels.** Blood vessels within the CNS tissue proper, as contrasted with vessels in the leptomeninges.

**Leptomeninges.** The two inner layers of the meninges: the pia mater and arachnoid mater. The leptomeninges are within the CNS; the blood vessels in the leptomeninges exhibit blood–brain barrier (BBB) properties. The overlying dura mater is outside the CNS and contains blood vessels that lack BBB properties.

**Leukocyte adhesion molecule.** A cell surface protein that mediates the interaction of circulating immune cells with the endothelium. These include intercellular adhesion molecules (ICAMs), vascular cell adhesion molecule 1 (VCAM1), P-selectin and E-selectin.

**Müller glia.** A glial cell type in the retina that is a major source of the blood–retina barrier-regulating molecule Norrin.

**Mural cells.** The outer cellular component of blood vessels, encompassing pericytes (on microvessels) and vascular smooth muscle cells (on large vessels).

**Receptor-mediated transcytosis.** The process by which binding of a macromolecule ligand to a cell surface receptor leads to ligand internalization within a vesicle (endocytosis), trafficking across the cell and release (exocytosis) at the opposing cell membrane.

**Vascular basement membrane.** A layer of extracellular matrix on the abluminal surface of blood vessels, which contributes to vessel structure/stability and serves as a scaffold for signaling molecules. In large vessels, a perivascular space separates an endothelial/mural basement membrane and an astrocyte endfoot-associated parenchymal basement membrane. At the capillary level, these merge into a single vascular basement membrane.

**Vascular organotypicity.** The specialization of vascular properties to meet the demands of an organ. The blood–brain barrier is an example of endothelial vascular organotypicity.

Several mechanisms contribute to the restrictive nature of the BBB, such as: highly complex and abundant tight junctions between adjacent endothelial cells; lack of small pores called fenestrae (see Glossary, Box 1); and an extremely low number of vesicles compared to the endothelial cells of other organs (Brightman and Reese, 1969; Nagy et al., 1984; Palade, 1961; Reese and Karnovsky, 1967) (Table 1). Additional restrictive mechanisms include efflux transporters that extrude a wide array of lipophilic molecules that diffuse into the endothelial plasma membrane, low expression of leukocyte adhesion molecules (see Glossary, Box 1) that limits CNS access of circulating immune cells, and the glycocalyx, a luminal glycoprotein/glycolipid coating that regulates access of molecules and cells to the endothelial surface. The BBB also regulates substance exchange with the brain by expressing solute carriers, such as the glucose transporter GLUT-1, that facilitate nutrient uptake and waste clearance in a highly specific manner, machinery for macromolecule uptake via receptor-mediated transcytosis (see Glossary, Box 1), metabolic enzymes that alter molecular transport/diffusion, as well as signaling mediators (Table 1). Collectively, these BBB properties – which serve as examples of vascular organotypicity (see Glossary, Box 1) – limit non-specific access of blood components to the CNS and facilitate the delivery of necessary substances, thus contributing to the tight regulation of CNS environment required for neural signaling.

**Table 1.** Molecular markers of blood–brain barrier phenotype

Family/type	Molecules	References
Tight junctions	Claudin 5^+^ (*Cldn5*)	Nitta et al. (2003)
	Occludin^+^ (*Ocln*)	Hirase et al. (1997)
	LSR^+^ (*Lsr*)	Sohet et al. (2015)
	ZO-1^+^ and ZO-2^+^ (*Tjp1* and *Tjp2*)	Schulze et al. (1997); Watson et al. (1991)
	Tricellulin^+^ (*Marveld2*)	Iwamoto et al. (2014)
Fenestra related	Plasmalemma vesicle-associated protein (PLVAP)^–^ (MECA-32^–^) (*Plvap*)	Hallmann et al. (1995)
Vesicle trafficking related	PLVAP^–^ (MECA-32^–^) (*Plvap*)	Hallmann et al. (1995)
	MFSD2A^+^ (*Mfsd2a*)	Andreone et al. (2017); Ben-Zvi et al. (2014)
Glycocalyx	Hyaluronan^high^	Larsen et al. (2025 preprint)
Solute carriers	GLUT-1^+^ (*Slc2a1*)	Cornford et al. (1994); Pardridge et al. (1990)
	LAT-1^+^ (*Slc7a5*)	Boado et al. (1999)
Efflux transporters	P-glycoprotein (P-gp)^+^ (*Abcb1a/b*)	Cordon-Cardo et al. (1989); Daneman et al. (2010); Petropoulos et al. (2010); Qin and Sato (1995); Schinkel et al. (1994); Thiebaut et al. (1989)
	Breast cancer resistance protein (BCRP)^+^ (*Abcg2*)	Cisternino et al. (2004); Cygalova et al. (2008)
Immune cell-adhesion molecules	Intercellular adhesion molecule 1 (ICAM1)^low^ and vascular cell-adhesion protein 1 (VCAM1)^low^ (*Icam1* and *Vcam1*)	Henninger et al. (1997)
Receptor-mediated transcytosis	Transferrin receptor (TfR)^+^ (*Tfrc*)	Jefferies et al. (1984)
	Low density lipoprotein receptor (LDLR)^+^ (*Ldlr*)	Dehouck et al. (1997)
Metabolism	Alkaline phosphatase^+^ (*Alpl*)	Vorbrodt et al. (1986)
	Dopamine decarboxylase^+^ monoamine oxidase^+^ (*Ddc*, *Maoa/b*)	Bertler et al. (1964)
Signaling	LEF1^+^ (*Lef1*)	Stenman et al. (2008)
	ZIC3^+^ (*Zic3*)	Ben-Zvi et al. (2014); Sabbagh et al. (2018)

In the CNS, vascular endothelial cells, which are the only cells in contact with the circulating blood, form the barrier under homeostatic conditions. Additionally, the vascular basement membrane (see Glossary, Box 1), formed by endothelial-, mural- and astrocyte-derived proteins, also contributes to some aspects of barrier function (Kutuzov et al., 2018). The astrocytic glia limitans forms an additional barrier to immune cell infiltration in neuroinflammatory contexts, such as experimental autoimmune encephalomyelitis (EAE) (see Glossary, Box 1) (Agrawal et al., 2006). Thus, once cells or molecules cross the endothelium, the vascular basement membrane and glia limitans serve as additional impediments to CNS uptake. Other cell types, including pericytes, astrocytes, Müller glia, neurons, perivascular fibroblasts and immune cells, also play important roles in regulating the endothelial BBB phenotype; these cells are collectively termed the neurovascular unit (Grimes et al., 2025; Neuwelt, 2004; Schaeffer and Iadecola, 2021). Additionally, many BBB properties are plastic and dynamically regulated by physiological cues such as neural activity and circadian rhythms (Pulido et al., 2020; Zhang et al., 2021). While the core aspects of BBB phenotype are present across different CNS regions and along the entire vascular tree, the BBB exhibits molecular and functional heterogeneity across both of these dimensions (Blanchette et al., 2025; Vanlandewijck et al., 2018). In addition to the BBB, which is only one of several barriers that maintain CNS homeostasis, choroid plexus epithelial cells and arachnoid barrier (see Glossary, Box 1) cells form blood–cerebrospinal fluid (CSF) barriers, while retinal pigment epithelial cells establish an outer blood–retinal barrier (Ban and Rizzolo, 2000; Brightman and Reese, 1969; Dani et al., 2021; Derk et al., 2023; DeSisto et al., 2020; Huang et al., 2009; Kniesel and Wolburg, 1993; Nabeshima et al., 1975). Barriers isolating circumventricular organs (CVOs; see Glossary, Box 1), regions of the brain that perform sensory and secretory functions, and lack a BBB (Oldfield and McKinley, 2015), from the CSF and surrounding CNS tissue are incompletely understood but likely involve specialized ependymal cells, including tanycytes, and the extracellular matrix (Kuczynski-Noyau et al., 2024; Langlet et al., 2013).

The BBB has clinical relevance both as an impediment to CNS drug delivery and because its dysfunction is a contributor to neurological diseases (Lajoie and Shusta, 2015; Profaci et al., 2020). Notably, recent discoveries of molecular mechanisms of BBB development open new avenues for BBB-targeting therapeutic strategies and motivate continued efforts to delineate BBB developmental processes (Ding et al., 2023; Martin et al., 2022). In this Review, we discuss current knowledge of BBB development, including the timing of the main stages underlying BBB development and the induction of endothelial BBB phenotype by extrinsic, tissue-derived signals. We also summarize some of the key cell–cell interactions and signaling pathways that regulate acquisition of BBB properties, highlighting the crucial role of Wnt/β-catenin signaling and endothelial co-receptors that confer ligand specificity. Finally, we review established and emerging experimental techniques for studying BBB development and highlight unanswered questions in the field.

## Timing of BBB development

The function of the BBB depends on numerous molecular specializations of endothelial cells that contribute to distinct functional properties, which develop in a coordinated but asynchronous fashion. Although BBB development is a continuous process, we find it helpful to conceptualize as three phases: the first that occurs concurrent to initial CNS angiogenesis; a second phase occurring immediately after initial angiogenesis; and a third phase that occurs during expansion and maturation of the vascular network (Fig. 1B).

### Phase 1: initial BBB development alongside CNS angiogenesis

CNS vascularization occurs exclusively via angiogenesis: the development of new blood vessels from existing vessels. The initial phase of BBB development occurs alongside the initial phase of CNS angiogenesis, which takes place at different developmental time points in different CNS tissues. In the mouse, brain and spinal cord angiogenesis begins at approximately embryonic day (E) 9.5, when sprouts originating from the perineural vascular plexus invade the neural tube, first in caudal (posterior) regions then proceeding rostrally (anterior) (Daneman et al., 2010; Nagase et al., 2005; Nakao et al., 1988). In the mouse retina, angiogenesis occurs postnatally, with vessels originating from the optic nerve head and migrating radially toward the retinal periphery (Gerhardt et al., 2003). In zebrafish hindbrain, which is frequently used to study CNS vascular development due to its stereotyped vascular architecture and ease of imaging, angiogenesis begins at ∼30-36 h post-fertilization (hpf), with sprouts originating from the primordial hindbrain channels forming the central arteries (Fujita et al., 2011; Ulrich et al., 2011; Umans et al., 2017). In all of the aforementioned cases, endothelial cells immediately begin to express the glucose transporter GLUT-1, accompanied by reduced neural tissue GLUT-1 expression, with further increases in BBB GLUT-1 abundance occurring over time (Bolz et al., 1996). The bicellular tight junction protein claudin 5 is expressed in vascular endothelial cells throughout the embryo prior to CNS angiogenesis (Collins et al., 2012), but expression of the tricellular tight junction protein LSR (angulin 1) is induced in a caudal-to-rostral gradient between E11.5 and E13.5 (Sohet et al., 2015). Vascular sprouts also rapidly lose their fenestrae, which are not observed after E13 in mouse (Stewart and Hayakawa, 1994).

### Phase 2: further BBB development after initial angiogenesis

Although some molecular specializations of the BBB develop concurrently with angiogenesis, increased restrictiveness to ion and molecule permeability occurs during the second phase. In anesthetized rats, transendothelial electrical resistance, a measure of restrictiveness to ions, increases from ∼300 Ω cm^2^ at mid-gestation to >1000 Ω ·cm^2^ at late gestation, without significant further increase postnatally (Butt et al., 1990). The increase during gestation is likely driven by increasing abundance and branching (complexity) of tight junction networks, which connect adjacent endothelial cell membranes (Kniesel et al., 1996). Similarly, permeability to high molecular weight tracers, such as 10 kilodalton dextran, decreases between E13.5 and E15.5 in mouse (Ben-Zvi et al., 2014). A similar decrease in dextran permeability occurs in the zebrafish midbrain between 4 and 5 days post-fertilization (dpf), while the hindbrain vasculature is already restrictive to dextran at 3 dpf (O'Brown et al., 2019), and a decrease in small molecule permeability is observed between 8 and 10 dpf (Fleming et al., 2013). In the postnatal day (P)3-P5 mouse, vessels in the central retina exhibit reduced permeability compared to angiogenic vessels in the peripheral retina (Chow and Gu, 2017). These effects correlate with a reduction in endothelial vesicle density (Bauer et al., 1993) and decreased expression of plasmalemma vesicle-associated protein (PLVAP), a protein that forms fenestral and caveolar diaphragms (Daneman et al., 2010; Stan et al., 1999). Efflux transporters, which remove certain small, lipophilic substances from the endothelial plasma membrane, are also induced during this second phase. Although the mRNA encoding the efflux transporter P-glycoprotein can be detected in E10.5 mouse CNS vasculature (Qin and Sato, 1995), mRNA- and protein-level expression of P-glycoprotein increases between E15.5 and E18.5, which correlates with decreased accumulation of P-glycoprotein substrate in the CNS (La Manno et al., 2021; Petropoulos et al., 2010) (Fig. 1C). The function of another efflux transporter, breast cancer resistance protein (BCRP), similarly increases during late gestation (Cygalova et al., 2008). Finally, the immune cell adhesion molecule ICAM1 is initially expressed in CNS vasculature, but is subsequently downregulated and is undetectable at late embryonic timepoints (Daneman et al., 2010). Together, these findings indicate a coordinated progression that ensures transition of CNS endothelial cells to a highly specialized barrier phenotype.

### Phase 3: BBB development during expansion and maturation of the vascular network

After its initial formation, the vascular network in the CNS undergoes substantial remodeling, including additional sprouting angiogenesis and the refinement of arterioles, capillaries and venules. In the rodent brain, these processes occur during the early postnatal period (from birth to ∼3 weeks of age) (Coelho-Santos et al., 2021; Rowan and Maxwell, 1981), which also coincides with the maturation of the BBB. At the ultrastructural level, between birth and 6 weeks of age, there is a reduction in interendothelial junctional clefts, which are gaps between adjacent endothelial cell membranes that may be a route for paracellular molecule/ion exchange, and a thickening of the vascular basement membrane (Stewart and Hayakawa, 1987). Expression of the tight junction protein occludin increases dramatically between P8 and P70 in the rat (Hirase et al., 1997), and P-glycoprotein expression also increases postnatally (Daneman et al., 2010). Several genes expressed in the adult BBB, including *Slco1a4*, which encodes an organic anion transporter, and *Itih5*, which encodes a protease inhibitor, exhibit limited expression at all embryonic timepoints, indicating that transcriptional programs that confer BBB phenotype continue to act postnatally (La Manno et al., 2021) (Fig. 1C). These observations demonstrate that specific aspects of BBB function develop much later than those canonically associated with initial CNS angiogenesis, although this later phase is understudied compared to the first two phases of BBB development.

## Extrinsic signals control BBB development

Initial evidence of extrinsic, tissue-derived signals controlling BBB phenotype comes from heterotopic grafting experiments. Svendgaard and colleagues transplanted rat iris tissue, which normally lacks BBB properties, into the forebrain (Svendgaard et al., 1975). Vessels in the transplanted iris tissue grafts lacked the BBB-characteristic abilities to decarboxylate l-DOPA, a precursor of the neurotransmitter dopamine, and exclude neurotoxic 6-hydroxydopamine from the tissue. Conversely, CNS tissue transplanted adjacent to the iris contained vessels with BBB properties, indicating that signals from the CNS environment may be sufficient to induce BBB characteristics in non-CNS vasculature (Svendgaard et al., 1975). However, these experiments did not conclusively demonstrate that BBB-like vessels originate from the host tissue rather than the graft, which can be accomplished using the quail–chick chimera technique wherein nuclear morphology permits unambiguous discrimination between graft and host cells (Le Douarin, 1973). Stewart and Wiley (1981) transplanted somites, mesodermal tissue segments of vertebrate embryos, from quail into the brain ventricle of chick embryos. The transplanted somite grafts contained vessels that are host (chick)-derived, which grow into the mesodermal graft from the CNS. These resulting vessels lack alkaline phosphatase activity, are permeable to Trypan Blue (a synthetic dye used to assess BBB permeability) and have abundant vesicles – all of which are characteristic of a non-BBB phenotype. Conversely, quail brain tissue transplanted into chick coelom contains host-derived vessels that exhibit BBB properties (Stewart and Wiley, 1981) (Fig. 1D). Furthermore, neural tissue is capable of inducing expression of the BBB-enriched HT7 antigen (that was later identified as the membrane glycoprotein basigin) in quail–chick chimeras (Ikeda et al., 1996; Seulberger et al., 1992). Together, these experiments demonstrate that BBB properties are not intrinsic to CNS vasculature and are instead induced and maintained by signals from CNS tissue. The rapid loss of BBB properties in cultured CNS endothelial cells provides additional support for the importance of continued tissue-derived signals in maintaining BBB properties (Rauh et al., 1992; Sabbagh and Nathans, 2020). In the following sections, we summarize these tissue-derived signals and the cell types from which they are derived.

### Wnt/β-catenin signaling regulates BBB development

**Fig. 2.**
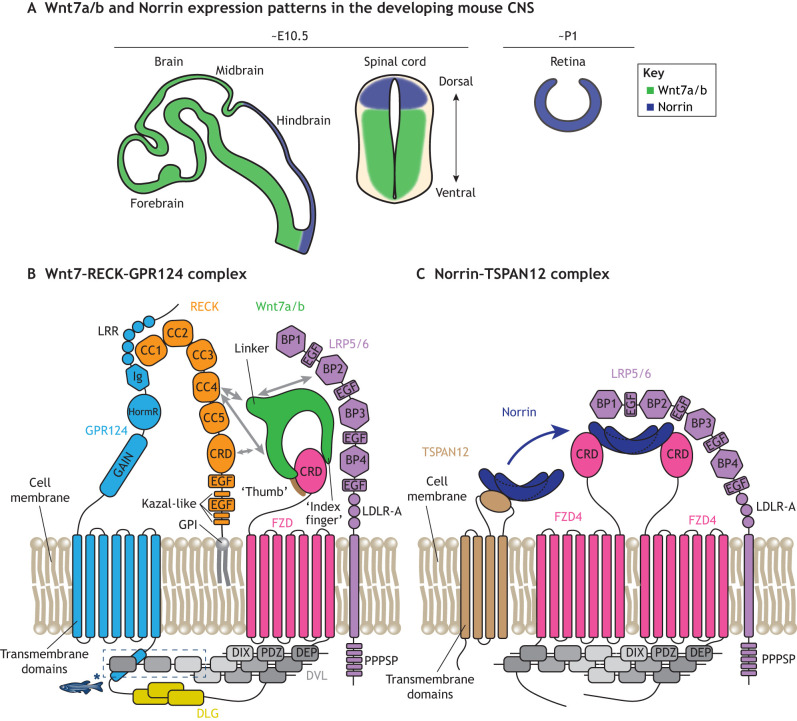
**Wnt/β-catenin signaling in BBB development.** (A) Expression patterns of the ligands Wnt7a and/or Wnt7b (Wnt7a/b) and Norrin in the developing mouse. Schematics of the brain and spinal cord at approximately embryonic day (E) 10.5 and retina at approximately postnatal day (P) 1 are shown. (B,C) Schematics of the endothelial receptor complexes that transduce Wnt7 (B) and Norrin (C) signaling. Schematics based on previously published structural data (Chang et al., 2015; Janda et al., 2012; Qi et al., 2023; Qian et al., 2024; Shen et al., 2015) and biochemical studies. Gray arrows in B indicate that the interaction of RECK with Wnt7 may be mediated by RECK CC4 and/or CRD and the Wnt7 N-terminal domain and/or linker, and that the Wnt7 linker may additionally interact with LRP5/6. BP, beta-propeller; CC, dicysteine domain, also known as a cysteine knot (CK) domain; CRD, cysteine-rich domain; DEP, Dishevelled, Egl-10, Pleckstrin domain; DlX, Disheveled and Axin domain (a core interaction module within the cytoplasmic scaffold protein Disheveled); DLG, discs large homolog; DVL, disheveled; EGF, epidermal growth factor-like domain; FZD, frizzled; GAIN, G protein-coupled receptor autoproteolysis-inducing domain; GPI, glycosylphosphatidylinositol; HormR, hormone receptor domain; Ig, immunoglobulin-like domain; LDLR-A, low-density lipoprotein receptor class A domain; LRR, leucine-rich repeat; PDZ, Postsynaptic density protein-95, Discs large tumor suppressor, Zonula occludens-1 domain; PPPSP, proline-proline-proline-serine-proline motif; TSPAN12, tetraspanin 12. Asterisk indicates that the intracellular interaction between disheveled and GPR124 occurs in zebrafish but not in the mouse.

Neural progenitor cells are the predominant cell type in the embryonic CNS at the time of initial angiogenesis and barriergenesis. Neural progenitor-derived vascular endothelial growth factor (VEGF) is required for angiogenesis (Hogan et al., 2004). Further, cultured neural progenitors can improve BBB properties in cultured brain endothelial cells compared to monocultured endothelial cells (Weidenfeller et al., 2007). During the period of initial CNS angiogenesis (∼E9.5-11.5 in mouse), neural progenitors express Wnt ligands, with some expressed broadly and others restricted to specific spatial domains. Notably, the ligands Wnt7a and Wnt7b exhibit broad expression in the forebrain, midbrain, ventral hindbrain and ventral spinal cord (Daneman et al., 2009; Parr et al., 1993; Stenman et al., 2008) (Fig. 2A). Endothelial cells in the developing CNS exhibit canonical Wnt/β-catenin signaling activity, and β-catenin signaling is required for CNS angiogenesis and barriergenesis: Mice deficient in endothelial β-catenin or Wnt7a/Wnt7b exhibit varying degrees of CNS vascular dysfunction, including reduced vascular density, lack of endothelial GLUT-1 expression, aberrant expression of PLVAP and hemorrhage (Daneman et al., 2009; Liebner et al., 2008; Stenman et al., 2008).

#### Norrin activates endothelial β-catenin signaling in a region-dependent manner

In the developing retina, endothelial β-catenin signaling is activated by Norrin, a protein secreted by Müller glia (see Glossary, Box 1) and potentially other cell types such as astrocytes (Ye et al., 2009). Although Norrin lacks sequence homology with Wnts, it specifically binds frizzled 4, a member of the Wnt receptor family, but does not interact with other frizzled (FZD) receptors (Smallwood et al., 2007). Norrin production is stimulated by glutamatergic neural activity and activates β-catenin signaling to regulate angiogenesis and blood–retinal barrier (BRB) (see Glossary, Box 1) formation via a mechanism that requires receptor endocytosis (Biswas et al., 2024; Wang et al., 2012; Xu et al., 2004; Ye et al., 2009; Zhang et al., 2017). [In this Review, we use the term BRB to refer to the inner BRB. An outer BRB (between choroid and retina) is formed by the retinal pigment epithelium.] FZD4 knockout mice and zebrafish exhibit retinal vascular defects, similar to the effect of disrupting endothelial β-catenin signaling in the developing brain and spinal cord (Caceres et al., 2019; Wang et al., 2012). FZD4 knockout mice also exhibit BBB leakage in the cerebellum, olfactory bulb and spinal cord, but not in cerebral cortex, suggesting other FZD receptors such as FZD1, FZD6 and/or FZD8 may function redundantly in the cerebral cortex (Daneman et al., 2009; Wang et al., 2012). Norrin–FZD4 signaling is also required for development of the inner ear vasculature, which, although outside the CNS, forms a blood–labyrinth barrier analogous to the BBB/BRB (Rehm et al., 2002; Sakagami et al., 1982; Xu et al., 2004). Similarly, in addition to retinal expression, Norrin is also highly expressed in the dorsal hindbrain and spinal cord, regions with relatively less Wnt7a/Wnt7b expression compared to other CNS regions, during embryogenesis (Ye et al., 2011) (Fig. 2A). Concordantly, mice lacking Norrin exhibit BBB leakage in the cerebellum but not in other brain regions (Wang et al., 2012). The Wnt co-receptors LRP5 and LRP6, which are members of the low density lipoprotein receptor-related protein family, are also required for BBB formation, with the relative importance of LRP5 versus LRP6 varying across CNS regions (Zhou et al., 2014). Together, these results demonstrate that β-catenin signaling is required for BBB development across the CNS, and the relative role of different neural tissue-derived ligands and endothelial receptors vary in a region-specific manner (Table 2).

**Table 2.** Summary of vascular phenotypes associated with loss of function mutations in components of the β-catenin signaling pathway

Gene	Phenotype in mice	Phenotype in zebrafish	References
*Wnt7a*	None apparent	*wnt7aa*: reduced hindbrain CtAs *wnt7ab*: none apparent	Daneman et al. (2009); Martin et al. (2022)
*Wnt7b*	In forebrain and spinal cord: reduced vascular density; vascular malformations; reduced BBB function	*wnt7ba* and *wnt7bb*: none apparent	Daneman et al. (2009); Martin et al. (2022)
*Wnt7a*+*Wnt7b*	In forebrain and spinal cord: reduced vascular density; vascular malformations (more severe than Wnt7b loss of function); reduced BBB function	Unknown	Daneman et al. (2009)
*Norrin* (*Ndp*)	In retina: reduced vascular density; vascular malformations; reduced BRB function In cerebellum: reduced BBB function	Unknown	Wang et al. (2012); Xu et al. (2004); Ye et al. (2009)
*Fzd4*	In retina, cerebellum, olfactory bulb, and spinal cord: reduced BBB function	In retina: reduced ventral vessel remodeling	Caceres et al. (2019); Wang et al. (2012); Xu et al. (2004); Ye et al. (2009)
*Reck*	In forebrain and spinal cord: reduced vascular density; reduced BBB function; hemorrhage	No hindbrain CtAs	Chandana et al. (2010); Cullen et al. (2011); Vanhollebeke et al. (2015)
*Gpr124* (*Adgra2*)	In forebrain and spinal cord: reduced vascular density; reduced BBB function; hemorrhage (more severe than RECK loss of function	No hindbrain CtAs	Kuhnert et al. (2010); Vanhollebeke et al. (2015); Zhou and Nathans (2014)
*Tspan12*	In retina: reduced vascular density; vascular malformations; reduced BRB function	Unknown	Junge et al. (2009)

BBB, blood–brain barrier.

#### RECK, GPR124 and TSPAN12 potentiate endothelial β-catenin signaling

Wnt ligands are broadly expressed throughout the vertebrate embryo, function in numerous developmental processes and display promiscuous binding to FZD receptors (Dijksterhuis et al., 2015; Lickert et al., 2001; Takada et al., 1994). This raises a natural question: why do endothelial cells in non-CNS organs not acquire BBB properties in response to these Wnts? One possibility is that other CNS-specific signals might be required to induce BBB-specific characteristics. However, ectopic expression of Wnt7a is sufficient to partially confer BBB phenotype to non-CNS vessels in mouse embryos (Stenman et al., 2008), suggesting that Wnt7 ligands may be uniquely capable of activating endothelial β-catenin signaling. This specificity is conferred by the G-protein-coupled receptor GPR124 and the membrane-anchored glycoprotein RECK, as mice and zebrafish lacking GPR124 or RECK phenocopy the CNS vascular deficits observed in Wnt7a/Wnt7b mutants (Anderson et al., 2011; Chandana et al., 2010; Cullen et al., 2011; Kuhnert et al., 2010; Vanhollebeke et al., 2015). Together, GPR124 and RECK form a Wnt7-specific receptor complex on endothelial cells (Cho et al., 2017; Posokhova et al., 2015; Ulrich et al., 2016; Vallon et al., 2018; Vanhollebeke et al., 2015; Zhou and Nathans, 2014) (Fig. 2B).

Wnt7a and Wnt7b, but none of the other 17 Wnts or Norrin, bind to RECK in a manner mediated by a linker region on Wnt7, which is distal to the FZD-binding interface and exhibits high sequence divergence from other Wnts (Cho et al., 2019a; Eubelen et al., 2018). Structural data, however, indicate that the Wnt7 N-terminal domain interacts with RECK and that Wnt linkers interact with LRP6 (Qi et al., 2023; Tsutsumi et al., 2023). Together, this suggests that a weaker Wnt7 linker-mediated interaction with RECK may precede the establishment of Wnt7 N-terminal–RECK and Wnt7 linker–LRP5/6 interactions. Both the cysteine-rich domain and a dicysteine domain of RECK have been implicated in Wnt7 binding, and RECK additionally stabilizes the monomeric, active form of Wnt7 (Eubelen et al., 2018; Vallon et al., 2018). The presence of Wnt7 ligand further enhances GPR124 binding to RECK (Cho et al., 2017; Eubelen et al., 2018). In zebrafish, the intracellular domain of GPR124 potentiates β-catenin signaling by recruiting scaffolding proteins Disheveled (DVL) that bind the Frizzled intracellular domain, whereas in mice, an intracellular domain-independent mechanism appears to play a more prominent role (America et al., 2022; Cho et al., 2019b; Yuki et al., 2024). Thus, the RECK–GPR124–FZD complex permits endothelial cells to gain BBB properties in response to CNS-derived Wnt7 ligands in both mice and zebrafish (Fig. 2B).

In the mouse retina, both global and endothelial cell-specific tetraspanin 12 (TSPAN12) knockouts phenocopy Norrin and FZD4 knockouts: they exhibit malformed superficial and intermediate vascular plexi, complete absence of the deep vascular plexus, aberrant PLVAP expression and BRB leakage (Junge et al., 2009; Zhang et al., 2018). TSPAN12 interacts with both Norrin and FZD4 via its large extracellular loop (Lai et al., 2017): it directly binds Norrin in a manner competitive with Norrin-LRP5/6 binding, suggesting that TSPAN12 enhances endothelial β-catenin signaling by concentrating Norrin in the vicinity of FZD4 prior to the association of Norrin with FZD4–LRP5 (Bruguera et al., 2025) (Fig. 2C). Thus, there is a conserved requirement for co-receptors that bind specific extracellular signals and potentiate endothelial β-catenin signaling to induce BBB properties, although the identity of these co-receptors varies across CNS regions (Table 2). Notably, the Wnt7–RECK–GPR124 and Norrin–TSPAN12 complexes provide a foundation for therapeutics that re-activate developmental β-catenin signaling to restore BBB and BRB function in disease (Ding et al., 2023; Martin et al., 2022).

#### Other Wnt/β-catenin signaling regulators

In addition to some of the widely studied factors discussed above, several other molecules and pathways also interact with Wnt/β-catenin signaling to regulate BBB development. The ubiquitously expressed deubiquitinase USP9X is required for BBB development and likely functions by directly interacting with β-catenin to prevent its degradation (Lei et al., 2025). Several molecules transcriptionally induced by β-catenin also exert positive and negative feedback control on Wnt/β-catenin signaling intensity and timing. One such molecule is APCDD1, a membrane protein that binds and sequesters Wnts, including Wnt7a (Hsieh et al., 2023). *Apcdd1*-knockout mice exhibit accelerated BRB formation and transiently increased vascular density, suggesting that APCDD1 negatively regulates β-catenin signaling (Mazzoni et al., 2017). FGFBP1 is a secreted extracellular matrix protein that potentially binds Wnt ligands, and *Fgfbp1*-knockout mice exhibit BBB defects, suggesting that this molecule effects positive feedback on Wnt/β-catenin signaling (Cottarelli et al., 2020). The receptors DR6 and TROY, members of the superfamily of tumor necrosis factor receptors, are also induced downstream of β-catenin signaling and regulate CNS-specific angiogenesis and BBB development (Tam et al., 2012).

β-Catenin signaling is differentially regulated along the arteriovenous axis (see Glossary, Box 1) of the CNS vasculature. The neural guidance factor netrin 1 (Ntn1) receptor, Unc5b, is required for BBB maintenance (Boyé et al., 2022) and is highly enriched at the arterial end of the vascular tree in both brain and retina (Furtado et al., 2024; Vanlandewijck et al., 2018). Early postnatal loss of Ntn1 or endothelial Unc5b in mouse causes a loss of BRB phenotype in retinal arterioles and capillaries, but not in venules, whereas loss of endothelial Lrp5 causes an opposing venular/capillary phenotype. The Ntn1–Unc5b interaction activates β-catenin signaling and regulates a cohort of genes similar to that controlled by Norrin-induced β-catenin signaling (Furtado et al., 2024). Thus, although the upstream regulators of BRB gene expression and function differ along the vascular tree, the resulting downstream phenotypes appear similar.

#### Transcriptional regulation downstream of β-catenin

At the transcriptional level, the three transcription factors in the subfamily of Sox F transcription factors, Sox7, Sox17 and Sox18, are regulated by β-catenin signaling in CNS endothelial cells and display distinct patterns of expression along the vascular tree, with Sox7 enriched in arterioles, Sox17 enriched in arteries and arterioles, and Sox18 depleted in veins (Corada et al., 2013; Zhou et al., 2015). During embryonic and early postnatal development, Sox17 is also reciprocally required for endothelial β-catenin signaling activity. Additionally, over the course of development, a gradual decline in endothelial β-catenin signaling is accompanied by an increase in the proportion of Sox17-expressing endothelial cells. Furthermore, early postnatal loss of endothelial Sox17 leads to widespread BBB leakage and aberrant PLVAP expression, which can be rescued by β-catenin activation (Corada et al., 2019). These results suggest that, over time, the transcriptional machinery regulating the BBB genetic program shifts from high levels of β-catenin to a synergistic combination of Sox17 and low levels of β-catenin. The zinc-finger transcription factor Zic3 is also BBB specific, regulated by β-catenin signaling and capable of inducing BBB genes *in vitro* (Hupe et al., 2017; Wang et al., 2012). Epigenomic analyses also suggest that Zic3 may broadly regulate BBB-specific genes (Sabbagh et al., 2018), highlighting the need for additional investigation of Zic3 function in BBB development.

### Astrocytes as regulators of BBB development

Astrocytes are considered a potential regulator of BBB development because they are found only in neural tissue, possess specialized endfoot structures that are closely associated with blood vessels, and improve BBB properties of endothelial cells *in vitro* (Maxwell et al., 1987; Tao-Cheng et al., 1987; Tontsch and Bauer, 1991). When implanted into the anterior chamber of rat eyes, astrocytes form aggregates that exhibit lower dye permeability than meningeal cell aggregates and the adjacent iris, suggesting that astrocytes are sufficient to induce BBB properties. Furthermore, astrocytes implanted on chick chorioallantoic membrane similarly exhibit low dye uptake (Janzer and Raff, 1987). However, some studies showed that astrocyte grafts on the iris and chorioallantoic membrane are poorly vascularized, suggesting that low dye uptake may be a result of hypovascularization rather than BBB function (Holash et al., 1993). Additionally, the isolation method employed in the earlier work (Janzer and Raff, 1987) may have yielded a mixed population, including astrocytes, progenitor cells, pericytes and fibroblasts, which would preclude conclusions about the function of a specific cell type. Grafted cells also exhibit a reactive (or activated) phenotype, as evidenced by glial fibrillary acidic protein (GFAP) expression, that is likely distinct from their homeostatic phenotype during development. In mice, astrocyte generation occurs at late embryonic timepoints (beginning around E14 in the spinal cord and around E16 in the cortex), with further generation, maturation and endfoot association with blood vessels occurring postnatally, well after many molecular and functional attributes of the BBB are established (Daneman et al., 2010; Magavi et al., 2012; Tien et al., 2012). Thus, while astrocytes may be sufficient to regulate BBB properties, they are not required for initial barriergenesis.

Although astrocytes are not essential for BBB induction during embryonic angiogenesis, they have roles in aspects of BBB maturation and maintenance (Table 3). Astrocyte endfoot coverage of blood vessels dramatically increases during early postnatal development in the mouse (Freitas-Andrade et al., 2023), and between 3 and 9 dpf in zebrafish (Gall et al., 2025). FGF2-knockout mice, which display delayed astrocyte endfoot association with blood vessels, do not exhibit increased BBB permeability to sucrose or endogenous plasma proteins, indicating that early postnatal association of endfeet with blood vessels is dispensable for BBB function (Saunders et al., 2016). Similarly, early postnatal loss of astrocyte *Hmgb1* causes endfoot abnormalities and an increased number of large endothelial vesicles, without apparent effects on endothelial junctions or tracer permeability (Freitas-Andrade et al., 2023). On the other hand, while Wnt7 and Norrin ligands are produced by neural progenitor cells during embryogenesis, their expression shifts to neurons and glia in the adult CNS (Vanlandewijck et al., 2018; Zeisel et al., 2018). Inhibiting astrocyte Wnt secretion in adult mice leads to endfoot abnormalities, as well as increased BBB permeability and endothelial vesicle density (Guérit et al., 2021). Astrocyte-derived angiotensin II and angiopoietin 1, proteins with diverse roles in vascular development and function, are also implicated in BBB function (Lee et al., 2003; Wosik et al., 2007). Astrocyte-derived Sonic hedgehog (Shh) has also been proposed as a regulator of BBB development: Tie2-Cre-mediated conditional deletion of the Shh receptor Smoothened leads to BBB leakage and reduced tight junction protein expression, while pharmacological inhibition of Smoothened reduces CNS immune cell infiltration in EAE (Alvarez et al., 2011). Subsequent work, however, suggests that astrocytes themselves, and not endothelial cells, exhibit hallmarks of active Shh signaling in the postnatal mouse CNS (Wang et al., 2021; Zeisel et al., 2018). Conditional deletion of Smoothened in astrocytes causes BBB leakage in hypothalamus and spinal cord, but not in other CNS regions (Wang et al., 2021). While astrocytes may play a role in the maturation phase of BBB development (Table 3), the underlying molecular mechanisms remain incompletely defined.

**Table 3.** Mediators of astrocyte and pericyte functions in blood–brain barrier development

Gene and expression pattern	Modification	Phenotype	References
*Evi* (Wntless, *Wls*; ubiquitously expressed)	Adult loss of function in astrocytes	Increased BBB permeability; increased number of endothelial vesicles; impaired astrocyte endfoot polarization	Guérit et al. (2021)
*Hmgb1* (ubiquitously expressed)	Postnatal loss of function in astrocytes	Reduced astrocyte endfoot vessel coverage; increased number of endothelial large (macropinocytotic) vesicles;	Freitas-Andrade et al. (2023)
*Agt* (angiotensinogen, precursor of angiotensins; selectively expressed in astrocytes)	Global loss of function	Disorganized BBB occludin	Wosik et al. (2007)
*Smo* (smoothened; ubiquitously expressed)	Loss of function in astrocytes	In hypothalamus and spinal cord: increased BBB permeability; increased number of endothelial vesicles	Wang et al. (2021)
*Pdgfb* (platelet-derived growth factor B, PDGFB; selectively expressed in endothelial cells) or *Pdgfrb* (PDGF receptor β, PDGFRβ; selectively expressed in mural cells)	Global loss of function	No CNS pericytes; increased BBB permeability; increased number of endothelial vesicles; BBB tight junction ultrastructural abnormalities; increased BBB expression of leukocyte adhesion molecules; increased abundance of leukocytes in CNS; perinatal lethality	Daneman et al. (2010); Hellström et al. (1999); Levéen et al. (1994); Lindahl et al. (1997); Soriano (1994)
*Pdgfrb* (selectively expressed in mural cells)	*Pdgfrb*^F7^ mutant with impaired signaling to downstream kinases	Reduced CNS pericyte coverage; increased BBB permeability during development	Daneman et al. (2010); Tallquist et al. (2003)
*Pdgfb* (selectively expressed in endothelial cells)	*Pdgfb*^ret^ mutant with impaired binding to heparin sulfate proteoglycans	Reduced CNS pericyte coverage; increased BBB permeability; increased number of endothelial vesicles; impaired astrocyte endfoot polarization	Armulik et al. (2010); Török et al. (2021)
*Notch3* (selectively expressed in mural cells)	Global loss of function	Zebrafish: reduced CNS pericyte coverage; increased BBB permeability; hemorrhages Mouse: loss of arterial vascular smooth muscle cells; increased BBB permeability	Henshall et al. (2015); Wang et al. (2014)
*Rbpj* (ubiquitously expressed)	Postnatal loss of function in mural cells	In the brain: hemorrhage; vascular malformations; abnormal pericyte transcriptional phenotype without change in pericyte coverage; reduced vascular smooth muscle cell coverage In the retina: reduced CNS pericyte and vascular smooth muscle cell coverage; hemorrhage; vascular malformations	Diéguez-Hurtado et al. (2019); Nadeem et al. (2020)
*Foxf2* (selectively expressed in endothelial cells and pericytes)	Global loss of function	Increased CNS pericyte proliferation; increased number of endothelial vesicles; increased BBB permeability; hemorrhage	Reyahi et al. (2015)
*Tgfbr1* (TGFβ receptor 1, ALK5; ubiquitously expressed)	Loss of function in mural cells	Reduced CNS pericyte coverage; hemorrhage; increased endothelial proliferation; abnormal basement membrane	Dave et al. (2018)
*Smad4* (ubiquitously expressed)	Loss of function in endothelial cells	Reduced CNS pericyte coverage; increased BBB permeability; hemorrhage	Li et al. (2011)
*Foxc1* (selectively expressed in mural cells, endothelial cells and fibroblasts)	Loss of function in mural cells	Increased CNS pericyte proliferation; microhemorrhages; increased endothelial proliferation	Siegenthaler et al. (2013)
CD146 (*Mcam*) (selectively expressed in mural cells, oligodendrocytes and embryonic endothelial cells)	Global loss of function, loss of function in endothelial or mural cells (see text)	Reduced CNS pericyte coverage; increased BBB permeability; reduced BBB claudin 5 expression; BBB tight junction ultrastructural abnormalities	Chen et al. (2017)
Vitronectin (selectively expressed in pericytes)	Global loss of function	In retina and cerebellum: increased BBB permeability; increased number of endothelial vesicles	Ayloo et al. (2022)

BBB, blood–brain barrier.

### Pericytes as regulators of BBB development

Similar to astrocytes, pericytes are widely used to improve the BBB properties of cultured endothelial cells (Berezowski et al., 2004; Dohgu et al., 2005; Hori et al., 2004; Nakagawa et al., 2009). However, unlike astrocytes, pericytes are present on nascent CNS blood vessels (Hellström et al., 1999) and thus are poised to regulate the initial stages of BBB development. Endothelium-derived platelet-derived growth factor B (PDGFB) signals through PDGF receptor β (PDGFRβ) on pericytes to mediate their recruitment to the endothelium, and embryos depleted of *Pdgfb* or *Pdgfrb* lack pericytes and exhibit perinatal lethality (Hellström et al., 1999; Levéen et al., 1994; Lindahl et al., 1997; Soriano, 1994). Mice carrying a *Pdgfrb* allele with seven tyrosine-to-phenylalanine mutations that impair PDGFRβ signaling to downstream kinases remain viable but exhibit reduced pericyte coverage in the CNS, further indicating the importance of PDGFB–PDGFRβ signaling in regulating pericyte development (Tallquist et al., 2003). During embryonic development, these pericyte-deficient mice exhibit increases in BBB permeability, endothelial vesicle number and endothelial expression of immune cell adhesion molecules, correlating with higher number of immune cells in the CNS, while endothelial claudin 5 and GLUT-1 levels remain normal (Daneman et al., 2010). Similarly, mice in which PDGFB binding to heparan sulfate proteoglycans (major components of the extracellular matrix) is disrupted exhibit reduced pericyte coverage, a transcytosis-dependent increase in BBB permeability, disrupted astrocyte endfoot polarization, increased presence of immune cells in the CNS and hyper-susceptibility to EAE (Armulik et al., 2010; Török et al., 2021). Together, these findings suggest that pericytes are required for the development and function of the BBB and the broader NVU.

Several additional genetic factors regulating pericyte development and function also have concomitant effects on BBB development. First, loss of mural cell (see Glossary, Box 1) *Notch3* or activin receptor-like kinase 5 (Alk5) lead to varying degrees of reduced CNS pericyte coverage and BBB dysfunction (Dave et al., 2018; Wang et al., 2014). On the other hand, loss of forkhead box F2 (FOXF2) leads to an increase in the number of pericytes, but also causes BBB dysfunction (Reyahi et al., 2015). Additionally, loss of endothelial *Smad4*, which encodes a highly conserved intracellular mediator of TGFβ superfamily signaling, reduces endothelial N-cadherin expression, which impairs a homotypic interaction with pericyte N-cadherin, leading to reduced pericyte coverage and BBB dysfunction (Li et al., 2011). Finally, mice deficient in CD146, a cell-adhesion molecule with both endothelial and mural cell expression during development and mural cell-restricted expression in adults (Chen et al., 2017; Vanlandewijck et al., 2018), exhibit reduced pericyte coverage, increased BBB permeability to a variety of molecular tracers and reduced endothelial claudin 5 expression. Furthermore, conditional knockout of CD146 in mural cells largely phenocopies the global knockout, but endothelial-specific knockout also causes increased permeability and decreased claudin 5. In this context, CD146 may influence pericyte recruitment and BBB development via an interaction with PDGFRβ (Chen et al., 2017).

Collectively, the above findings suggest that pericytes influence BBB development via mechanisms parallel to and at least partially independent from β-catenin signaling (Table 2): β-catenin signaling induces GLUT-1, increases claudin 5 and suppresses PLVAP, while pericytes inhibit transcellular transport and immune cell adhesion and/or transmigration, and also suppress PLVAP. Although the exact molecular mechanisms remain unknown, limited available data suggest that multiple mechanisms may contribute to pericyte-mediated regulation of BBB development (Table 3). One hypothesis is that CNS pericytes express CNS-specific molecules that signal to endothelial cells. Alternatively, the same molecules may be produced by pericytes throughout the body but affect CNS endothelial cells differently because of the higher abundance of pericytes in the CNS. Finally, a third hypothesis posits that the same pericyte-derived molecules may be interpreted differently by CNS endothelial cells, potentially influenced by β-catenin or other CNS-specific signaling. It is known that loss of the transcription factor Foxc1, which is enriched in CNS mural cells compared to mural cells of other organs, leads to focal BBB leakage and vessel hypertrophy (Muhl et al., 2020; Siegenthaler et al., 2013; Vanlandewijck et al., 2018); however, this phenotype is less severe than the CNS-wide BBB dysfunction observed in pericyte-deficient mice, suggesting that Foxc1-independent mechanisms are likely major contributors to BBB regulation. On the other hand, vitronectin, a serum and extracellular matrix glycoprotein, is highly expressed by mouse pericytes across multiple organs (Muhl et al., 2020; Vanlandewijck et al., 2018), but its loss results in BBB leakage and an increased number of endothelial vesicles in the retina and cerebellum (Ayloo et al., 2022). However, the reason behind the regionally heterogeneous requirement for vitronectin is unknown.

### Additional extrinsic signals regulate BBB development and function

In addition to Wnt/β-catenin signaling and signals derived from astrocytes and pericytes, several additional tissue-derived factors also contribute to development of the BBB. Retinoic acid can induce BBB properties in cultured endothelial cells, and pharmacological inhibition of retinoic acid receptor β impairs BBB function in mouse embryos (Mizee et al., 2013). In the neocortex, retinoic acid suppresses expression of Wnt inhibitors by neural progenitors and thereby positively regulates BBB formation, whereas in other regions such as the striatum and thalamus, it functions as an endothelial cell-autonomous negative regulator of β-catenin signaling (Bonney and Siegenthaler, 2017; Bonney et al., 2016, 2018). In the retina, impaired endothelial cell retinoic acid signaling causes delayed angiogenesis without affecting BRB function (Como et al., 2023). Reelin (Reln), a secreted glycoprotein that regulates neuronal migration, also regulates BBB development: mice lacking *Reln*, or with endothelial cell-specific deletion of *Dab1* (an intracellular mediator of Reln signaling) exhibit BBB leakage and reduced astrocyte endfoot coverage, suggesting an endothelial cell-autonomous response to Reln. Remarkably, cortical layering is also disrupted in endothelial *Dab1* knockouts, highlighting the importance of bidirectional neural-vascular interactions in CNS development (Segarra et al., 2018).

## Complementary cellular and molecular mechanisms underlying BBB development

Many of the molecular markers of BBB phenotype discussed above (Table 1) directly link to BBB function. For example, GLUT-1 mediates glucose uptake and claudin 5 forms tight junctions. Other molecules highly enriched at the BBB compared to peripheral vasculature, such as docosahexaenoic acid transporter MFSD2A (Nguyen et al., 2014), likely contribute to BBB function through more complex regulatory or signaling functions. Some studies have shown that *Mfsd2a*-deficient mice and zebrafish exhibit increased BBB and BRB leakage driven by increased transcytosis, and suggest that MFSD2A suppresses transcytosis by delivering docosahexaenoic acid to the endothelial plasma membrane, which impairs the formation of caveolae (Andreone et al., 2017; Ben-Zvi et al., 2014; Chow and Gu, 2017; O'Brown et al., 2019). On the other hand, several other studies have reported a lack of BBB leakage in *Mfsd2a*-knockout mice (Andaloussi Mäe et al., 2020; Wong et al., 2016). These contradictory findings indicate that the function of MFSD2A in BBB development may require further clarification.

Additional endothelial cell-expressed molecules also regulate BBB development: for example, the glycosylphosphatidylinositol (GPI)-linked protein Doppel, encoded by *Prnd*, is transiently expressed during CNS angiogenesis and regulates the activity of receptor tyrosine kinases, and *Prnd-*knockout mice exhibit angiogenesis defects and BBB leakage (Chen et al., 2020). Similarly, the transmembrane protein FLRT2 is selectively expressed in venous endothelium and mice with early postnatal loss of endothelial *Flrt2* have angiogenesis defects, BBB leakage and abnormal BBB tight junctions (Llaó-Cid et al., 2024). Notably, while the above molecules regulate both angiogenesis and BBB function, mice deficient for *Flvcr2*, which encodes a BBB-enriched choline transporter (Cater et al., 2024), exhibit defective angiogenesis without apparent dysfunction of the BBB (Santander et al., 2020). Thus, while some molecules preferentially influence angiogenesis or barriergenesis, a large number of signals co-regulate these processes, suggesting that they are tightly coupled in CNS endothelial cells.

Beyond crucial signaling molecules, biochemical and biophysical aspects of cell–matrix interactions also regulate BBB development. The CNS vascular basement membrane is composed of collagen IV, laminins, fibronectin, nidogen and heparan sulfate proteoglycans produced by endothelial cells, pericytes and astrocytes. Several of these molecules and their endothelial integrin receptors are developmentally regulated and required for CNS vascular function (Barber and Lieth, 1997; Dong et al., 2002; Jeanne et al., 2015; Milner, 2002; Risau and Lemmon, 1988; Sorokin et al., 1997). In particular, endothelial cells, mural cells and astrocytes produce a diverse repertoire of laminins, many of which are required for BBB development and function (Menezes et al., 2014; Nirwane et al., 2024; Thyboll et al., 2002). Conditional knockout of laminin γ1 in astrocytes results in increased BBB permeability and pericyte abnormalities (Yao et al., 2014). Similarly, conditional knockout of laminin γ1 in pericytes leads to an age-dependent increase in BBB permeability, loss of pericyte coverage and loss of astrocyte endfoot polarization (Gautam et al., 2016, 2020). Together, these observations suggest a crucial role for basement membrane laminin in regulating BBB development and maintenance. Additionally, neuron-derived Spock1, which regulates proper formation of the vascular basement membrane, is also required for BBB development (O'Brown et al., 2023). Many of these basement membrane proteins are not CNS specific and are required for vascular integrity throughout the body. There are, however, several BBB-enriched genes that encode basement membrane proteins, such as *Spock2* and *Vwa1*, and endothelial β-catenin signaling regulates a cohort of such BBB-enriched genes in the embryonic mouse forebrain (Jensen et al., 2019; Munji et al., 2019), motivating future work to define their functions.

The extracellular matrix also serves as an important site for the retention of signaling molecules involved in BBB development (e.g. PDGFB). During CNS development, the extracellular matrix also retains latent TGFβ, which is activated by binding to α_V_β_8_ integrins expressed by neural cells. Subsequent signaling through endothelial TGFβ receptor 2 negatively regulates angiogenic sprouting and promotes vascular integrity (Allinson et al., 2012; Arnold et al., 2012; De et al., 2022; Mu et al., 2002; Nguyen et al., 2011; Proctor et al., 2005). In tip cells, Notch mediates expression of neuropilin 1, which binds to neural integrins and negatively regulates TGFβ activation, thus promoting the angiogenic phenotype (Aspalter et al., 2015; Fantin et al., 2013; Hirota et al., 2015).

Finally, mechanical forces may contribute to CNS vascular development. Endothelial cells throughout the body are exposed to blood flow, and the associated shear stress is sensed and transduced by a PECAM1–VE-cadherin–VEGFR2 complex that activates integrins, the mechanosensitive ion channel Piezo1 and other understudied mechanisms (Ranade et al., 2014; Tzima et al., 2005). These processes regulate endothelial alignment, sprouting, pruning, permeability and arteriovenous identity (reviewed by Campinho et al., 2020). Endothelial mechanosensation also has non-cell autonomous effects, e.g. endothelial Piezo1 is required for brain pericyte proliferation in zebrafish (Zi et al., 2024). Additionally, in cultured CNS endothelial cells, the lack of shear stress and corresponding downregulation of shear-responsive transcription factors KLF2 and KLF4 may be partially responsible for the observed loss of *in vivo*-like gene expression (Foreman et al., 2025; Sabbagh and Nathans, 2020). However, blood flow and endothelial expression of molecular mediators of shear stress signaling are not CNS specific; thus, any role for these processes in development of the BBB is likely mediated via interactions with CNS-specific signals.

## Development of BBB heterogeneity

All segments or ‘zones’ of the CNS vascular tree from arterioles to capillaries to venules exhibit BBB properties, although zone-specific attributes exist. Arteriolar endothelial cells have higher vesicle density than capillary and venular endothelial cells (Chow et al., 2020; Hanske et al., 2017), while postcapillary venules serve as the main sites for immune cell trafficking and therapeutic nanoparticle transport (Kucharz et al., 2021; Vajkoczy et al., 2001). Many BBB-associated genes, such as *Tfrc*, *Mfsd2a* and *Vcam1*, display zonated expression (Jeong et al., 2022; Vanlandewijck et al., 2018). While zone-specific mechanisms that control a similar cohort of BBB/BRB properties, such as differential Ntn1–Unc5b/Norrin–Lrp5 signaling and arterial Dll4–Notch signaling, have been identified (Furtado et al., 2024; Yang et al., 2020), further work is needed to understand the mechanisms that control the development of zone-specific BBB properties.

Broadly, the developmental mechanisms of BBB regional heterogeneity are not well understood. CVO endothelial cells lack β-catenin signaling and ectopic activation of β-catenin is sufficient to impart BBB properties, even in adult animals (Benz et al., 2019; Wang et al., 2019). In the area postrema, a CVO located in the brainstem, the secreted Wnt inhibitor WIF1 is required to maintain the non-BBB state (Wang et al., 2019). VEGF, TGFβ and retinoic acid signaling also regulate the non-BBB CVO endothelial phenotype (Anbalagan et al., 2018; Parab et al., 2023). There are subtle differences in endothelial phenotype between different CNS regions with a BBB, e.g. blood vessels in the postnatal mouse dentate gyrus exhibit higher dextran permeability than in adjacent regions (Choe et al., 2015). Characterization of transcriptomic heterogeneity of the BBB across nine regions of the CNS demonstrated that BBB enrichment of the retinoid transporter Stra6 in the nucleus accumbens shell (a region within the ventral striatum) is important for the function of this region, and is likely regulated by the interaction between dietary vitamin A and yet unknown local signals (Blanchette et al., 2025). Leptomeningeal vessels (see Glossary, Box 1; at the surface of the CNS) also possess BBB properties, but they reside in an environment distinct from that of intraparenchymal vessels (see Glossary, Box 1), which are located in the CNS interior. Therefore, further research is needed to comprehensively characterize the distinct properties and associated developmental mechanisms in the leptomeninges and parenchyma.

## Strengths and limitations of current approaches for studying BBB development

Mouse and zebrafish are the two most widely used model organisms for studying BBB development. Although zebrafish are more evolutionarily distant from human and mouse, current knowledge suggests marked conservation of BBB molecular properties and developmental mechanisms across most vertebrates (reviewed by O'Brown et al., 2018). Advantages of zebrafish as a research model include optical transparency of larvae, fast development and an increasing number of genetically engineered models, especially for live-imaging applications (Table 4). The mouse is advantageous given the wide number of genetically engineered models available and its relatively short evolutionary distance from human. For mouse models, the Cre-Lox system (Orban et al., 1992) is the gold standard for investigating the genetic/molecular mechanisms of BBB development, given that it permits cell type-specific manipulation of gene expression that can also be temporally controlled in the case of tamoxifen-inducible CreER drivers. A number of endothelial Cre and CreER drivers have been employed to study BBB development (Table 4); however, when using these models, careful consideration of other cell populations that express the Cre and floxed target genes is crucial. For example, the widely used Cdh5-CreER^T2^ is active in both CNS and non-CNS endothelial cells. Thus, if the floxed target gene is not CNS specific, peripheral effects may contribute to any observed phenotypes. Furthermore, while Slco1c1-Cre(ER^T2^) is expressed in CNS but not in non-CNS endothelial cells, it is also expressed in other cell types, including choroid plexus epithelial cells. BBB endothelial cells and choroid plexus epithelial cells share many genes and functions, so phenotypes associated with either anatomical site must be interpreted with caution.

**Table 4.** Genetic models for blood–brain barrier labeling and manipulation in mouse and zebrafish

Organism	Strain	Type and notes	Allele symbol	References
Mouse	Tie2-Cre	Cre (transgene insertion) Also expressed in hematopoietic cells; potential female germline activity	Tg(Tek-cre)1Ywa	Kisanuki et al. (2001)
	Tie2-GFP	GFP reporter (transgene insertion)	Tg(TIE2GFP)287Sato	Motoike et al. (2000)
	Cdh5-CreER^T2^	Cre (transgene insertion)	Tg(Cdh5-cre/ERT2)1Rha	Sörensen et al. (2009)
	VE-cadherin-GFP	VE-cadherin-GFP fusion (knock-in) Also expressed in leptomeningeal fibroblasts	Cdh5^tm9Dvst^	Winderlich et al. (2009) Mapunda et al. (2023)
	Pdgfb-CreER	Cre (transgene insertion)	Tg(Pdgfb-icre/ERT2,-EGFP)1Frut	Claxton et al. (2008)
	Tie2-Cldn5-GFP	Claudin 5-GFP fusion under control of Tie2 promoter/enhancer (transgene insertion)	N/A	Knowland et al. (2014)
	Cldn5-GFP	GFP reporter (transgene insertion)	Tg(Cldn5-GFP)#Cbet	Vanlandewijck et al. (2018)
	Slco1c1-Cre	Cre (transgene insertion) Also expressed in choroid plexus epithelium	Tg(Slco1c1-icre)1Mash	Lang et al. (2011)
	Slco1c1-CreER^T2^	Cre (transgene insertion) Also expressed in choroid plexus epithelium	Tg(Slco1c1-icre/ERT2)1Mash	Ridder et al. (2011)
	Bmx-CreER^T2^	Cre (transgene insertion) Selectively expressed in arterial endothelium	Tg(Bmx-cre/ERT2)1Rha	Ehling et al. (2013)
	Mfsd2a-CreER^T2^	Cre (knock-in) Enriched expression in capillary (versus arterial and venous) endothelium	Mfsd2a^em1(cre/ERT2)Bzsh^	Pu et al. (2016)
	BAT-gal	β-Galactosidase reporter of β-catenin signaling (transgene insertion)	Tg(BAT-lacZ)3Picc	Maretto et al. (2003)
	TOPGAL	β-Galactosidase reporter of β-catenin signaling (transgene insertion)	Tg(TCF/Lef1-lacZ)34Efu	DasGupta and Fuchs (1999)
	Rosa26 TCF/LEF-LSL-H2B-GFP	Cre-dependent H2B-GFP reporter of β-catenin signaling (knock-in)	Gt(ROSA)26Sor^tm12(Tcf/Lef-GFP*)Nat^	Cho et al. (2017)
Zebrafish	kdrl:EGFP	eGFP reporter (transgene insertion)	Tg(flk1:EGFP)^s843^	Jin et al. (2005)
	kdrl:mCherry	mCherry reporter (transgene insertion)	Tg(flk1:ras-cherry)^s896^	Chi et al. (2008)
	kdrl:cre	Cre (transgene insertion)	Tg(kdrl:Cre)^s898^	Bertrand et al. (2010)
	flk1:NLSmCherry	nuclear mCherry reporter (transgene insertion)	Tg(kdrl:NLS-mCherry)^is4^	Wang et al. (2010)
	plvap:EGFP	eGFP reporter (transgene insertion)	Tg(plvap:EGFP)^sj3^	Umans et al. (2017)
	glut1b:mCherry	mCherry reporter (transgene insertion)	Tg(slc2a1a:mCherry)^sj1^	Umans et al. (2017)
	fli1:EGFP	eGFP reporter (transgene insertion)	Tg(fli1:EGFP)^y1^	Lawson and Weinstein (2002)
	fli1a:GAL4FF	GAL4FF (transgene insertion)	Tg(fli1a:Gal4FF)^ubs4^	Zygmunt et al. (2011)
	fli1a:Myr-mCherry	mCherry reporter (transgene insertion)	Tg(fli1a:MYR-mCherry)^ncv1^	Kwon et al. (2013)
	7×TCFsiam:GFP	GFP reporter of β-catenin signaling (transgene insertion)	Tg(7xTCF-Xla.Siam:GFP)^ia4^	Moro et al. (2012)

This table includes a selection of genetic models. For additional discussion of endothelial Cre mouse models (see Assmann et al., 2016; Payne et al., 2018).

Parenterally administered molecular tracers, such as horseradish peroxidase, Evans Blue, biotin, sodium fluorescein, dextrans and cadaverine, are extensively used to measure and/or estimate BBB function. Their use for developmental studies requires special attention to injection volume as a fraction of embryonic blood volume. Large injection volumes may cause artifactual BBB leakage, a phenomenon that contributed to a historical controversy surrounding the permeability of the embryonic BBB (reviewed by Engelhardt, 2003 and Saunders et al., 2014). The tight association of endothelial cells, pericytes and, at later developmental timepoints, astrocyte endfeet, complicates molecular analysis of the BBB. Commonly employed microvessel isolation protocols generate a mixture of these cell types; therefore, it is important to interpret the resulting data in this light or employ a stringent method to isolate endothelial cells (e.g. fluorescence-activated cell sorting with negative selection for contaminant cell types). This issue is largely obviated by single cell technologies, which also permit improved understanding of heterogeneity, especially across the arteriovenous axis (Betsholtz, 2022). Single cell and/or nucleus RNA-sequencing data of the developing CNS vasculature, both from model organisms and human samples, also offer an unprecedented source of hypotheses related to the molecular mechanisms of BBB development and function (La Manno et al., 2021; Wälchli et al., 2024). However, a limitation of these techniques often includes the lack of spatial information. Emerging spatial transcriptomic methods, especially those that permit true single cell-level resolution, may facilitate improved understanding of neurovascular cell heterogeneity *in situ*.

Although *in vivo* imaging is widely used to study CNS vasculature in the developing zebrafish and adult mouse (Berthiaume et al., 2018; O'Brown et al., 2019; Umans et al., 2017), it has been applied to developing mouse only in a limited number of studies (Coelho-Santos et al., 2021). Additionally, adeno-associated viruses that target CNS endothelium enable more facile genetic studies of BBB function in adulthood (Körbelin et al., 2016; Krolak et al., 2022), but they have not been widely adopted for developmental studies. Further technological advances in the areas of *in vivo* imaging, viral targeting and spatial omics should enable improved understanding of complex spatial, temporal and molecular aspects of BBB development.

*In vitro* cultures of endothelial cells, pericytes, astrocytes and other neurovascular cell types, which are widely used to investigate cellular and molecular cues capable of regulating BBB properties (Janzer and Raff, 1987; Rubin et al., 1991; Tontsch and Bauer, 1991; Weidenfeller et al., 2007), have important advantages: they allow mechanistic analysis of single factors and are amenable to genetic manipulation. However, these techniques also have certain disadvantages: cell phenotype dramatically changes when removed from the tissue microenvironment, so results obtained *in vitro* may not be predictive of *in vivo* function. Furthermore, it is difficult to investigate developmental processes with terminally differentiated cells, but emerging methods for differentiating BBB-like endothelial cells from pluripotent or adult stem cells offer a potential system for *in vitro* developmental studies (Gastfriend et al., 2021; Porkoláb et al., 2024; Praça et al., 2019; Roudnicky et al., 2020). These technologies also permit examination of developmental mechanisms in human systems, which may aid in understanding the basis of human developmental disorders and species-based differences in BBB properties (Song et al., 2020; Syvänen et al., 2009; Vatine et al., 2016).

## Conclusions and unanswered questions

The BBB is a set of endothelial properties that collectively regulate CNS homeostasis. Thus, understanding BBB development requires insights into the development of these diverse endothelial properties, as well as their underlying regulatory mechanisms. Most of the literature to date relies on a limited number of phenotypes as markers of BBB function such as tracer permeability and expression of claudin 5, GLUT-1 and PLVAP. Although omic-based techniques have expanded the breadth of characterization possible at the gene and/or protein expression level, we believe that there is a need for expanded functional assessment of BBB properties, including efflux transport, solute transport, metabolism and interaction with the immune system across development. Furthermore, the maturation phase of BBB development, which likely involves β-catenin-independent molecular mechanisms (as discussed previously), should be the subject of further detailed investigation.

We do not fully understand the mechanisms underlying BBB plasticity, which refers to the ability of endothelial cells to switch between a BBB and non-BBB state, and vice versa. Specifically, during embryogenesis, although non-CNS endothelial cells can adopt BBB phenotype in response to ectopic Wnt/β-catenin signaling (Stenman et al., 2008), adult liver, lung and anterior pituitary (the non-CNS component of the pituitary gland) endothelial cells largely lack this competence (Munji et al., 2019; Wang et al., 2019). Conversely, the endothelial cells in CVOs appear to retain such competence across the lifespan (Benz et al., 2019; Wang et al., 2019). Additional knowledge of how the endothelial cell chromatin state varies across development, and mechanistic studies in which chromatin-modifying factors are manipulated, may advance understanding in this area.

There are many unanswered questions related to the identity of specific signals derived from pericytes and astrocytes that regulate BBB development. Furthermore, additional investigation of potential roles for other cell types in regulating BBB development is required. For example, microglia regulate BBB function in the context of injury and disease, closely interact with developing vasculature and control retinal vascular architecture (Dudiki et al., 2020; Haruwaka et al., 2019; Mondo et al., 2020). However, microglia are not required for BBB function in healthy adult mice (Profaci et al., 2024), but whether they exert transient developmental influences on the BBB remains unknown. We also believe that there is a substantial lack of understanding of the developmental mechanisms that drive BBB heterogeneity between CNS regions and along the vascular tree. It is also unclear how diet and environmental factors may influence BBB development and resulting BBB function. Finally, it would be useful to understand the extent to which molecular mechanisms underlying BBB development are conserved across other species. BBB dysfunction accompanies several neurological disorders, and re-activation of developmental Wnt/β-catenin signaling has therapeutic benefit in animal models of glioblastoma, stroke and retinopathy (Chidiac et al., 2021; Ding et al., 2023; Martin et al., 2022). Addressing the above questions related to BBB development will likely contribute to the generation of new strategies for modulating BBB functions to treat neurological diseases in the future.

## Supplementary Material

10.1242/dev.205134_sup1 Supplementary information
