# Supplementary figures and images for: Development of the blood–brain barrier
Source: Development. 2026 Jan 23;153(2):dev205134. doi: 10.1242/dev.205134 (PMC12863308; doi:10.1242/dev.205134)

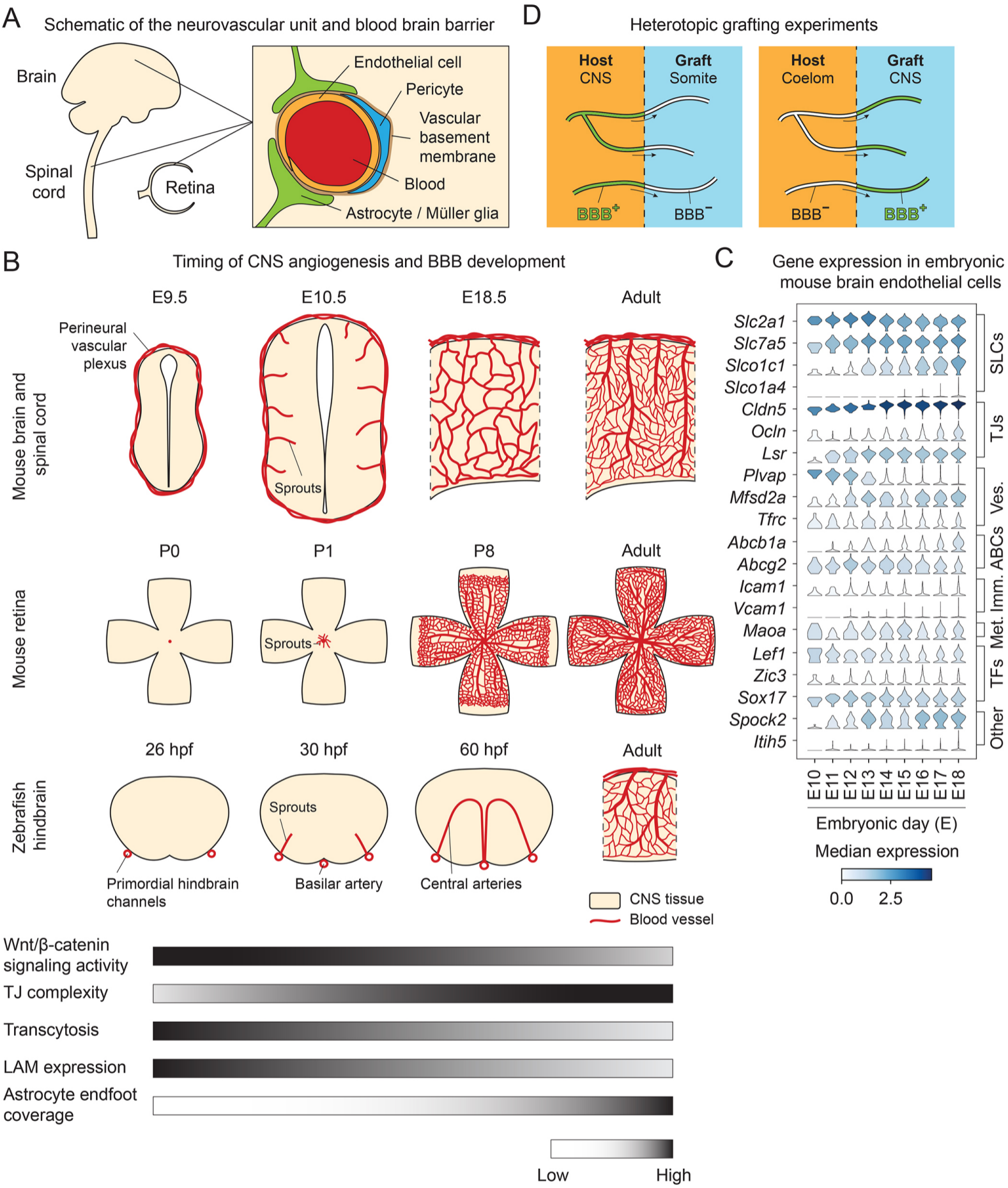

Fig. S1.

Supplement: Supplementary information [file develop-153-205134-s1.pdf]
